# Acidity promotes tumour progression by altering macrophage phenotype in prostate cancer

**DOI:** 10.1038/s41416-019-0542-2

**Published:** 2019-08-16

**Authors:** Asmaa El-Kenawi, Chandler Gatenbee, Mark Robertson-Tessi, Rafael Bravo, Jasreman Dhillon, Yoganand Balagurunathan, Anders Berglund, Naveen Vishvakarma, Arig Ibrahim-Hashim, Jung Choi, Kimberly Luddy, Robert Gatenby, Shari Pilon-Thomas, Alexander Anderson, Brian Ruffell, Robert Gillies

**Affiliations:** 1 Department of Pharmacology and Toxicology, Faculty of Pharmacy, Mansoura University, Mansoura, Egypt; 2 Department of Immunology, H. Lee Moffitt Cancer Center, Tampa, FL 33612 USA; 3 Department of Cancer Physiology, H. Lee Moffitt Cancer Center, Tampa, FL 33612 USA; 4 Department of Integrated Mathematical Oncology, H. Lee Moffitt Cancer Center, Tampa, FL 33612 USA; 5 Department of Anatomic Pathology, H. Lee Moffitt Cancer Center, Tampa, FL 33612 USA; 6 Department of Biostatistics, H. Lee Moffitt Cancer Center, Tampa, FL 33612 USA; 7 Department of Radiology, H. Lee Moffitt Cancer Center, Tampa, FL 33612 USA; 8 Department of Breast Oncology, H. Lee Moffitt Cancer Center, Tampa, FL 33612 USA

**Keywords:** Immune evasion, Cancer microenvironment

## Abstract

**Background:**

Tumours rapidly ferment glucose to lactic acid even in the presence of oxygen, and coupling high glycolysis with poor perfusion leads to extracellular acidification. We hypothesise that acidity, independent from lactate, can augment the pro-tumour phenotype of macrophages.

**Methods:**

We analysed publicly available data of human prostate cancer for linear correlation between macrophage markers and glycolysis genes. We used zwitterionic buffers to adjust the pH in series of in vitro experiments. We then utilised subcutaneous and transgenic tumour models developed in C57BL/6 mice as well as computer simulations to correlate tumour progression with macrophage infiltration and to delineate role of acidity.

**Results:**

Activating macrophages at pH 6.8 in vitro enhanced an IL-4-driven phenotype as measured by gene expression, cytokine profiling, and functional assays. These results were recapitulated in vivo wherein neutralising intratumoural acidity reduced the pro-tumour phenotype of macrophages, while also decreasing tumour incidence and invasion in the TRAMP model of prostate cancer. These results were recapitulated using an in silico mathematical model that simulate macrophage responses to environmental signals. By turning off acid-induced cellular responses, our in silico mathematical modelling shows that acid-resistant macrophages can limit tumour progression.

**Conclusions:**

This study suggests that tumour acidity contributes to prostate carcinogenesis by altering the state of macrophage activation.

## Background

Cancer initiation and progression involves complex cellular interactions of pre-malignant/malignant cells with immune, stromal cells and blood vessels. Levels of tissue oxygen, metabolic by-products, nutrients and hormones modulate these cellular interactions that, in turn, can regulate tumour progression.^1^ One important property of malignant cells is that they preferentially metabolise glucose into lactate even in the presence of oxygen—known as aerobic glycolysis or the “Warburg Effect”—which confers on them a growth advantage.^2^ Coupling elevated glycolysis with poor tumour perfusion leads to increased pericellular accumulation of organic acids (e.g. lactic acid) and reduced pH in extracellular spaces.^3^ Low pH induces the activity of proteolytic enzymes and can be toxic to surrounding stromal cells, leading to tissue remodelling and local invasion.^4,5^ It is also known to inhibit T cell-mediated immune surveillance,^6^ but the effect of tumour acidosis on the myeloid compartment within tumour is less well studied.

Tumours are infiltrated by populations of myeloid cells that regulate tumourigenesis through their ability to mediate immunosuppression, matrix remodelling, angiogenesis, local invasion and metastasis.^7,8^ In particular, infiltration by macrophages can promote tumour progression and poor outcome in solid malignancies when their presence is associated with a tumour-promoting phenotype reminiscent of interleukin (IL)-4-driven activation.^9^ The pro-tumour phenotype of these tumour-associated macrophages (TAMs) can be affected by several aspects of the tumour microenvironment (TME).^10^ These include cytokines and antibodies produced by lymphocytes and tumour-derived cytokines/chemokines that promote macrophage infiltration and polarisation.^11–13^ Abnormal metabolic factors can also aggravate the phenotype of these cells. For example, hypoxia augments the immunosuppressive ability of TAMs,^14^ while lactic acid induces tissue remodelling though expression of vascular endothelial growth factor (VEGF) and arginase I.^15^ Whether acidic pH, as an independent entity from lactate,^16^ alters macrophage polarisation within tumours is not clear, hence, we sought to investigate the impact of tumour acidosis on the phenotypic characteristics of macrophages in vitro using zwitterionic organic buffering agents. We then used a series of mouse models to correlate tumour progression with macrophage infiltration and to delineate the role of acidity in prostate cancer. We then reiterate our findings using an agent-based mathematical model that simulate how pH affects the ability of macrophages to control tumour growth.

## Methods

### Animal models

All mice were maintained in accordance with Institutional Animal Care and Use Committee (IACUC) standards followed by the Moffitt Cancer Research Center (Tampa, FL). Mice have free access to water and food, housed in pathogen-free cages containing wood shavings and bedding in a 12-h light/dark cycle, with controlled room temperature (RT). All animals and cell lines were male or male derived, respectively, since this study is mainly investigating prostate cancer. For bone marrow isolation, C57BL/6N (C57BL/6NHsd), aged 8–12 weeks, male mice were purchased from Envigo. For the subcutaneous prostate cancer model, mice were randomly assigned to experimental groups and then provided with 200 mM sodium bicarbonate in their drinking water (oral administration, mice have free access to water in their cages) starting on the fourth day prior to subcutaneous injections with 5 × 10^5^ TRAMP-C2 cells. This concentration of sodium bicarbonate in drinking water is well tolerated and provides the required buffering effect as described earlier.^17^ No anaesthesia, analgesia or surgical procedure were needed to administer the sodium bicarbonate solubilised in water. To ensure the health status of animals, mice weight and water consumption were monitored twice a week (data not included). Tumour growth was evaluated weekly by measurement of two perpendicular diameters of tumours with a digital calliper. Individual tumour volumes were calculated as volume = [*π*/6 × (width)^2^ × length]. To collect tumours, mice were euthanised using carbon dioxide inhalation in their home cages, followed by cervical dislocation to ensure death on the 35th–42nd day tumour cell postinjection. Solid tumours were then excised and processed for flow cytometric analysis and immunohistochemistry (IHC) as will be described later. Male transgenic adenocarcinoma of the mouse prostate (TRAMP) mice was obtained from The Jackson Laboratory. Male TRAMP spontaneously develops autochthonous prostate tumours following the onset of puberty due to the expression of the oncoprotein SV40 T antigen (TAg) under transcriptional control of the rat probasin promoter.^18^

### Cell lines

Male-derived murine TRAMP-C2 and TRAMP-C3 prostate cancer cell lines were purchased from ATCC, maintained and cultured according to their suggested protocols.

### Macrophage isolation, activation and cell culture protocols

Bone marrow-derived macrophages (BMDMs) were generated as described previously.^19,20^ In brief, bone marrow was flushed from femurs and tibias of male C57BL/6N mice and cultured for 6–7 days in complete macrophage medium (Dulbecco modified Eagle’s minimal essential medium supplemented with 10% foetal calf serum, 2% penicillin/streptomycin–glutamine) and 20 ng/ml macrophages colony-stimulating factor (M-CSF) at 37 °C. Pro-inflammatory macrophages were induced by exposing BMDMs to 50 ng/ml interferon (IFN)-γ and 10 ng/ml lipopolysaccharide (LPS) in complete macrophage medium. Anti-inflammatory macrophages were stimulated by exposure to 10 ng/ml IL-4 in complete macrophage medium.^19,21^ Control macrophages (M0) were cultured for the same period in medium alone. Prostate cancer-associated macrophages were induced by incubating BMDMs with 30% 72 h-conditioned medium from either TRAMP-C2 or TRAMP-C3 cell lines. To detect the effect of tumour microenvironmental acidity, macrophages were induced according the previous protocol but with further supplementation of media with the zwitterionic organic buffers PIPES and HEPES (25 mM each) and adjustment of the pH to either 7.4 or 6.8.^22^

### Antibodies, chemicals and kits

Recombinant mouse IFN-γ, M-CSF and IL-4 were obtained from R&D Systems. Sources of conjugated antibodies were as follows: inducible nitric oxide synthase (iNOS)-Alexa Fluor 488 (eBioscience), CD206-Alexa Fluor 647 (AbD Serotec), CD45-APC and MHCII-BV21 (BD Biosciences), and F4/80-PE, Ly6C-APC/Cy7 and CD11b-PE/Cy7 (BioLegend). Sources of unconjugated antibodies were as follows: anti-MRC1 (CD206) and anti-iNOS (Abcam). Source of chemical was as follows: Rhodamine Phalloidin (Life Technologies). Griess reagent (Promega) was used to measure nitrite level. Click-iT 5-ethynyl-2′-deoxyuridine (EdU) pacific blue flow cytometry assay kit (Life Technologies) was used to measure cell proliferation. Proteome profiler mouse cytokine array panel A or XL Cytokine Array ARY028 (R&D Systems) were used to detect change in level of cytokines in culture media. All reagents, kits and chemicals, unless otherwise stated, were used according to the manufacturers’ instructions. Other chemicals unless specified were purchased from Sigma-Aldrich.

### Real-time quantitative PCR (RT-qPCR) and NanoString profiling

RNA was extracted using the RNeasy Isolation Kit (Qiagen). RT-qPCR was then carried out using the iTaq Universal SYBER Green One-Step Kit (Bio-Rad) using primers specific for macrophage activation markers selected according to a previously published lists.^23–25^ Primers sequences are provided in (Supplemental Table S1). Results were normalised using 36B4 then expressed as fold change (FC) = 2^−ΔCt^, where ΔCt = (Ct_Target_ − Ct_36B4_).^24^ For gene expression analysis by NanoString nCounter, cell lysates were hybridised to the 770-gene murine PanCancer Immune Profiling Panel according to the manufacturer’s protocol (NanoString Technologies). Briefly, 10 µl of Ambion Cells-to-Ct buffer (Thermo Fisher Scientific) was added to a cell pellet and a 5.0-µl volume of lysate was hybridised to the NanoString reporter and capture probes in a thermal cycler for 16 h at 65 °C. Washing and cartridge immobilisation were performed on the NanoString nCounter PrepStation, and the cartridge was scanned at 555 fields of view on the nCounter Digital Analyser. The resulting RCC files containing raw counts were reviewed for quality and normalised in the NanoString nSolver analysis software v3.0, followed by exportation and analysis.

### Flow cytometry and sorting protocol

Cells were collected, washed and incubated at 4 °C in staining buffer (phosphate-buffered saline (PBS), 2% bovine serum albumin (BSA)) containing the indicated surface antibodies. For intracellular staining, cells were fixed, permeabilised and stained using the BD Cytofix/Cytoperm Fixation/Permeabilisation Kit (BD Biosciences) according to the manufacturer’s instructions. Cells were then washed with staining buffer and subsequently analysed. Data were recorded on a LSR II Flow Cytometer (BD Biosciences) and analysis completed using the FlowJo software. Additional details are included in supplemental materials and methods.

### Western blotting

Cell lysates with equal amounts of proteins (20–35 μg) were electrophoresed through 4–15% TGX Gel, then electrophoretically transferred to nitrocellulose membrane (Bio-Rad Laboratories). Membranes were then incubated with the specified antibodies diluted according to the manufacturer’s instructions. Membranes also were incubated with anti-α-tubulin or anti-glyceraldehyde 3-phosphate dehydrogenase as loading controls. Immunoreactive proteins were visualised with an appropriate peroxidase-conjugated secondary antibody.

### Confocal immunofluorescence

Macrophages cultured on chamber slides were washed twice with PBS, fixed in 3.8% formaldehyde for 20 min and permeabilised with 0.1% Triton X-100 for 5 min. Cells were washed twice with PBS, blocked with 2% BSA in PBS for 1 h and subsequently incubated with CD206 antibody (1:800) overnight at 4 °C. Cells were washed 3 times with PBS and incubated with appropriate fluorescent-labelled secondary antibodies at RT for 1 h. Images were visualised using Leica TCS SP8 laser scanning microscope (Leica Microsystems).

### Histology and IHC

The histological specimens were embedded in paraffin, sectioned (4-μm slices) and stained with haematoxylin & eosin. For IHC, slides were stained using a Ventana Discovery XT automated system (Ventana Medical Systems). Briefly, slides were deparaffinised on the automated system with EZ Prep solution (Ventana). Enzymatic retrieval method was used in Protease 1 (Ventana). The rabbit primary antibodies that react to F4/80, α-smooth muscle actin (α-SMA) and CD206 (all purchased from Abcam) were used at 1:400, 1:250 and 1:1200 dilutions, respectively, in Dako antibody diluent (Agilent) and incubated for 60 min. The Ventana OmniMap Anti-Rabbit Secondary Antibody was used for 8 min. The detection system used was the Ventana ChromoMap Kit, and slides were then counterstained with haematoxylin, followed by dehydrated and cover-slipping.

### Quantitative image analysis

Histology slides were scanned using the Aperio™ ScanScope XT with a ×200 (0.8 NA) objective lens at a rate of 5 min per slide via Basler tri-linear-array. For TRAMP-derived prostate tissue analysis, images and their meta-data were then imported into the Definiens Tissue Studio v4.0 suite. Each slide was then segmented into several tissue regions with stroma and gland being the main point of interest using the composer function in the software. The individual marker areas were then scored in terms of the intensity of F4/80, α-SMA and collagen. A pathologist (J.D.) was consulted to quality control that each tissue was correctly segmented into the regions of interest as shown in Supplemental Fig. S4F. For CD206 frequency, images and their meta-data were imported into the Definiens Tissue Studio v4.2 suite. Slides were then analysed by identifying individual cells using haematoxylin stain threshold and grown out to 2 μm. Cells were then identified by the expression of IHC markers CD206 and F4/80. The segmented images were imported in Definiens Developer v2.4, and image contrast was used first to separate the tumour section from the background. Next, a 25-pixel ring was segmented around the periphery of the tumour to represent the edge of the tumour. Finally, the distance (in μm) to the nearest edge of tumour pixel was calculated for each cell in the image. Since each tissue section is a different size and shape, each distance to the edge value was normalised per mm^2^ of tissue. The normalised distances were then subjected to histogram analysis to determine the percentage of cells that fall into 10 µm/mm^2^ bins representing areas of high macrophage abundance and higher acidity.

### The Cancer Genome Atlas (TCGA) Prostate Adenocarcinoma Data Set (PRAD) analysis

**Fig. 1:**
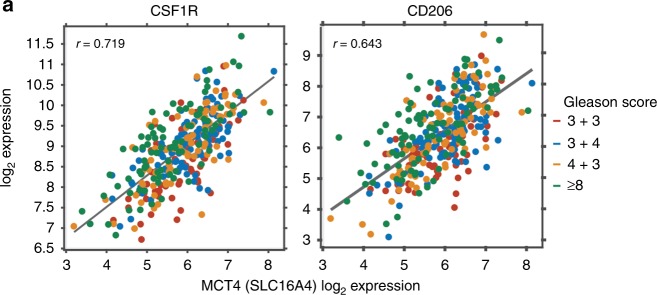
Macrophage infiltration correlates with MCT4 expression. Correlation between *CSF1R*/*CD206* and *MCT4* mRNA expression in early-stage patients (Gleason score = 3+3) and advanced prostate (Gleason score = 3+4, 4+3 and ≥8) retrieved from TCGA PRAD333: *R* = 0.719 and 0.643, respectively

The correlation of macrophage-related genes and glycolysis-related genes in a prostate cancer cohort was computed using level 3 gene expression estimates from the RNA-Sequencing in the TCGA PRAD database, extracted and hosted by Firehose DB (BROAD Institute, https://gdac.broadinstitute.org/). The expression estimates were derived using RSEM (Accurate transcript quantification from RNASeq) method.^26^ In Fig. 1a and S1A, the original level 3 Illumina HiSeq RNAseqV2 RSEM gene-level normalised mRNA expression data for TCGA PRAD was downloaded from the TCGA data portal in March of 2016 and log2 transformed, log2(*x* + 1). The 333 primary prostate tumours and associated clinical information, including reviewed Gleason score, were retrieved from the TCGA PRAD333 publication.^27^ Box and scatter plots were generated in MATLAB R2017a (MathWorks Inc.).

### Agent-based model

To examine the dynamics governing the interactions of macrophages and a metabolically aggressive tumour, we extended our previously published multiscale mathematical model that captures the complex spatiotemporal interactions of competing tumour cell phenotypes and microenvironmental selection forces, such as oxygen, glucose and acidosis.^28,29^ Macrophages were added to this model to explore their ability to control tumour growth within this complex and dynamic environment. While TAMs are often assumed to have an M2-immunosuppressive phenotype, in their M1 state they are able to phagocytise opsonised tumour cells and release inflammatory cytokines, thus potentially playing a role in tumour eradication.^30,31^ These in silico macrophages can consume tumour and necrotic cells, as well as release and bind macrophage-derived cytokines. Macrophage behaviour is modelled as a continuous phenotype from antitumour to pro-tumour like behaviours, determined by the local concentration of pH, pro- and anti-inflammatory cytokines and the number of tumour and necrotic cells being digested. Although T cells are a major component of the immune response in many solid tumours, prostate cancer tends to be immunologically cold and have minimal T cell infiltration.^32^ In the mathematical model, we do not include T cells in order to investigate the direct effects of macrophages on the tumour system in isolation.

**Fig. 2:**
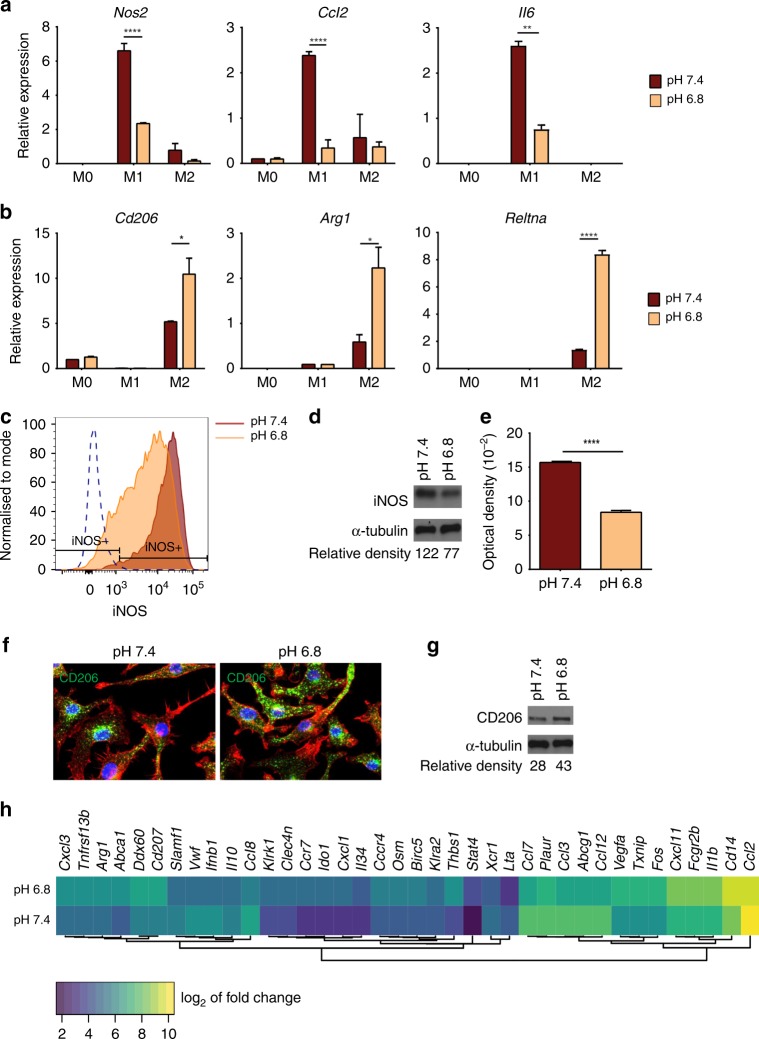
Extracellular acidosis alters macrophage activation in vitro. **a** mRNA expression of *Nos2*, *Ccl2* and *Il-6*. **b** mRNA expression of *Cd206*, *Arg1* and *Reltna* in bone marrow-derived macrophage stimulated for 24 h with lipopolysaccharide (LPS)/interferon (IFN)-γ (M1) or interleukin (IL)-4 (M2) or left untreated (M0) at either pH 7.4 or 6.8. Data are presented as mean ± SEM. Two-way analysis of variance was utilised for statistical analysis; *p < 0.05, **p < 0.01, ****p < 0.0001. **c** Expression of inducible nitric oxide synthase (iNOS) in LPS/IFN-γ activated macrophages at pH 7.4 or 6.8 using flow cytometry. **d** Western blot analysis of iNOS in LPS/IFN-γ activated macrophages at pH 7.4 and 6.8. α-Tubulin was used as a loading control. **e** Nitrite level in the supernatant of LPS/IFN-γ activated macrophages at pH 7.4 and 6.8, as measured by Griess reagent. Data are presented as mean ± SEM. Student’s *t* test was utilised for statistical analysis; ****p < 0.0001. **f** Confocal immunofluorescent analysis of CD206 expression in IL-4 activated macrophages at pH 7.4 or 6.8. CD206 (green), Phalloidin (red) and Dapi (blue). **g** Western blot analysis of CD206 expression in macrophages stimulated for 24 h with IL-4 (M2) at pH 7.4 and 6.8. α-tubulin was used as loading control. **h** Heatmap of the top differentially expressed genes (determined by *p* value and ranked by fold change) in LPS/IFN-γ activated macrophages at pH 7.4 or 6.8. nCounter PanCancer Immune Profiling that measures the expression of 770 genes was used to assess difference in gene expression (*n* = 2)

To calibrate the macrophage behaviour, we used the statistical package R^33^ to fit a linear model to specific gene expression data, in different ecological conditions, collected in the in vitro experiments described in Fig. 2h. Each linear model takes the form:

1 $$y_i = \alpha _i + \beta _ip + \gamma _ie + \delta _ipe,$$

where *y*_*i*_ is the observed expression level of gene *i*, *p* is the pH, *e* represents the ecological conditions and *α*_*i*_, *β*_*i*_, *γ*_*i*_ and *δ*_*i*_ are determined during the fitting. In the model, the value of *e* for each cell at each time point is calculated using the equation

2 $$e = - 0.5a - 0.5b + 0.5c + 0.5d,$$

where *a* is the local inflammatory cytokine concentration, *b* is the number of tumour cells phagocytised, *c* is the local anti-inflammatory cytokine concentration and *d* is the number of necrotic cells being phagocytised. During the fitting process, *e* is set to −1 for the inflammatory environment that promotes the extreme antitumour phenotype, while *e* is set 1 for the anti-inflammatory environment that induces the extreme pro-tumour phenotype. Thus each macrophage checks the local extracellular pH, pro- and anti-inflammatory cytokine levels and number of tumour and necrotic cells being digested and then adjusts each of the *i* phenotypic behaviours as dictated by the respective linear model. Further details are provided in supplemental materials and methods.

### Quantification and statistical analysis

Unless otherwise indicated, we used unpaired *t* test assuming Gaussian distribution and with Welch’s correction, where necessary. For multiple comparisons, two-way analysis of variance was used with Tukey’s correction, as appropriate. Unless otherwise reported, GraphPad PRISM 7 software was used for statistical analysis. In TCGA data analysis, a two-sided Mann–Whitney *U* test was used and median log_2_ fold change between the two groups was calculated. A significant change was defined when *p* < 0.05 and log_2_ fold change >0.585 (1.5× change). For the mathematical model, Mantel–Haenszel test in the R package “survival” was used.^34^ To identify changes in macrophage phenotype using NanoString, differentially expressed genes with *p* < 0.05 were ranked by fold change with a cut-off of 1.5 or 2.^35^ Statistical parameters, including the value of *n*, mean ± SEM and statistical significance, and the tests used are reported in the figures and/or figure legends.

## Results

### Macrophage infiltration correlates with MCT4 expression

Advanced stages of prostate cancer adopt a high glycolytic phenotype that correlated with poor prognosis.^36^ The consequent lactic acid production was shown to aggravate highly immunosuppressive microenvironment through shaping macrophage phenotype in lung cancer and melanoma.^15^ Based on that, we questioned whether highly glycolytic phenotype correlates with macrophage infiltration or phenotype in late-stage prostate cancer. Interestingly, analysing publicly available data of human prostate cancer revealed that *CSF1R* is expressed at higher levels in intermediate- and late-stage prostate cancers (Fig. S1A). In addition, *CSF1R* and the macrophage activation marker *CD206* correlated with the monocarboxylate lactate transporter *MCT4* (SLC16A4) in late-stage prostate cancer (Gleason score 3+4, 4+3 and ≥8), as shown in Fig. 1a and Supplemental Fig. S1B, C. Of note, MCT4 facilitates lactate efflux and preserves intracellular pH by co-transporting lactate and protons across the plasma membrane of highly glycolytic and/or acid-resistant cells.^16,37^ It is unknown whether the change in extracellular pH independent from changes in extracellular lactate concentration can modulate macrophage polarisation in prostate cancer.

### Extracellular acidosis alters macrophage activation in vitro

Macrophages are highly plastic immune cells that display a range of phenotypic and functional properties.^7,38^ To test whether an acidic tumour milieu can influence macrophage phenotype, we used zwitterionic buffer-based medium to stimulate BMDMs using IFN-γ/LPS and IL-4 for 24 h at pH 7.4 or 6.8. Under these conditions, acidic pH did not affect viability of stimulated macrophages at 24 h post-activation (Supplemental Fig. S2A). As seen in Fig. 2a, b, acidosis decreased the gene expression of the pro-inflammatory markers *Nos2*, *Ccl2* and *Il-6* in IFN-γ/LPS-polarised macrophages, while it increased the expression of anti-inflammatory markers *Cd206*, *Arg1* and *Reltna* in IL-4-polarised macrophages. Reduced iNOS protein levels were confirmed by flow cytometry and western blot (Fig. 2c, d). In line with the mRNA and protein expression data of iNOS, the level of nitrite in the culture media decreased, as shown in Fig. 2e. Enhanced *Cd206* expression in IL-4-polarised BMDMs was also confirmed by immunofluorescence and western blot (Fig. 2f, g). Multi-analyte profiling in culture medium from these incubations also revealed significant alterations in the release of many inflammatory cytokines and chemokines (Supplemental Fig. S2B, C). To expand these findings to other genes potentially involved in macrophage activation, we used NanoString profiling to assess the relative abundance of 770 cancer-and immune-related mRNAs. We observed that acidic pH increased the expression of a range of TAM-related genes (e.g. *Arg1*, *Cd14*, *Il1b*) as well as angiogenesis-associated genes (e.g. *Vegfa*, *Txnip*, *Thbs1*) in IFN-γ/LPS activated macrophages, in addition to a global decrease in the inflammation score (Fig. 2h, Supplemental Fig. S2D, E, and Supplemental Table S2). These results demonstrate that extracellular acidosis alters macrophage activation towards a phenotype reminiscent of TAMs in vitro.

### Extracellular acidosis enhances a tumour-promoting macrophage phenotype

**Fig. 3:**
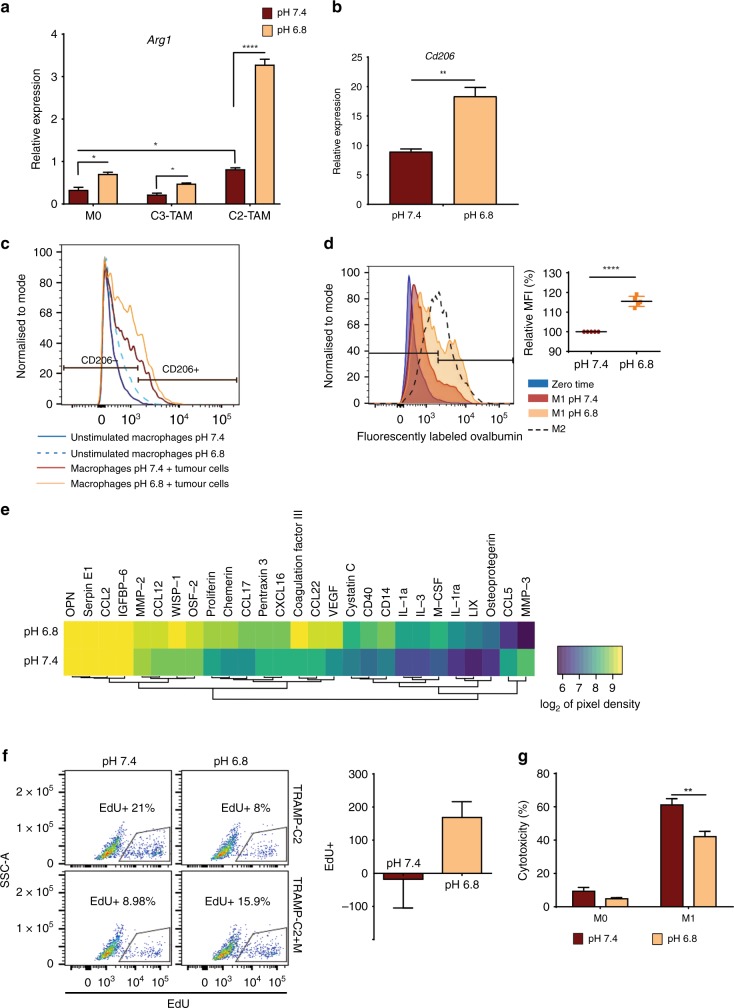
Extracellular acidosis enhances a tumour-promoting macrophage phenotype. **a** Relative mRNA level of *Arg1* in macrophages treated with 30% TRAMP-C2, TRAMP-C3 conditioned medium at either pH 7.4 or 6.8 or left untreated as control (M0). Data are presented as mean ± SEM. Two-way analysis of variance was utilised for statistical analysis; *p < 0.05, ****p < 0.0001. **b** Relative mRNA level of *Cd206* in macrophages directly co-cultured with TRAMP-C2 for 4 days at pH 7.4 or 6.8, then sorted and processed for RNA extraction. Data are presented as mean ± SEM. Student’s *t* test was utilised for statistical analysis; **p < 0.01. **c** Flow cytometric analysis of CD206 expression in macrophages incubated at pH 7.4 or 6.8 for 24 h, then either cultured alone or with TRAMP-C2 at pH 7.4 for another 24 h. F4/80 staining was used to gate out tumour cells. **d** Flow cytometric quantification of fluorescently labelled ovalbumin uptake in lipopolysaccharide (LPS)/interferon (IFN)-γ activated macrophages at either pH 7.4 or 6.8 for 24 h. Graph represents relative increase in fluorescently labelled ovalbumin uptake (*n* = 5). Data are presented as mean ± SEM. Student’s *t* test was utilised for statistical analysis; ****p < 0.0001. **e** Conditioned media from macrophage–tumour co-culture at pH 7.4 or 6.8 were processed for cytokine determination using the mouse XL cytokine array. Densitometric analysis was then done using the Image J software and pixel density was graphed as heatmap (*n* = 2). **f** TRAMP-C2 cells were co-cultured with or without macrophages in neutral or acidic medium for 24 h. Cells were then labelled with 5-ethynyl-2′-deoxyuridine (EdU) for 2 h, collected and processed for flow cytometric analysis. SSC vs. EdU fluorescence of TRAMP-C2 tumour cells in each culture condition was plotted. Fold change was calculated by dividing the EdU-incorporating cell count with macrophages by the corresponding values of tumour cells alone (*n* = 6). **g** Macrophages were activated with LPS/IFN-γ (M1) at pH 7.4 or 6.8 for 24 h or left unstimulated as M0. Differentially activated macrophages were then co-cultured with TRAMP-C3 cells and lactate dehydrogenase in the supernatants was measured 24 h later to estimate cytotoxicity. Data are presented as mean ± SEM. Two-way analysis of variance was utilised for statistical analysis; **p < 0.01

To examine whether extracellular acidity could alter activation status of TAMs, we first activated BMDMs with tumour cell-conditioned medium at either pH 7.4 or 6.8. At pH 7.4, TRAMP-C2-conditioned medium significantly increased the expression of *Arg1*. However, this effect was dramatically enhanced when the cells were activated at pH 6.8 (Fig. 3a). Similarly, co-culturing BMDMs with TRAMP-C2 and TRAMP-C3 at pH 6.8 augmented *Cd206* mRNA expression and protein levels in macrophages as measured by RT-PCR and flow cytometry, respectively (Fig. 3b, c, Supplemental Fig. S3A, B). BMDMs activated in acidic pH also increased the uptake of fluorescently labelled ovalbumin, a mannosylated ligand endocytosed mainly through CD206 (Fig. 3d). In addition, macrophage co-culture with TRAMP-C2 cells at acidic pH was associated with an increase in the release of inflammatory and angiogenic cytokines/chemokines (e.g. VEGF, CD14, M-CSF) known to be involved in tumour progression (Fig. 3e).

We next evaluated whether the phenotypic shift in macrophages would alter their function in vitro. TRAMP-C2 cells were incubated in acidic or neutral media in the presence or absence of non-polarised macrophages for 24 h, and tumour cell proliferation was measured via EdU uptake after gating out F4/80^+^CD11b^+^ macrophages. As shown in Fig. 3f, either acidic conditions or co-culture with unstimulated macrophages (pH 7.4) reduced tumour cell proliferation. In contrast, co-culturing with macrophages reversed the negative effect of acidic pH, resulting in a two-fold increase in proliferation. The total number of cells was unchanged during the relative short period of the experiment (Supplemental Fig. S3C). IFN-γ/LPS-stimulated macrophages are cytotoxic due to NO release; however, they lose their cytotoxic ability when activated at low pH (Fig. 3g). Acidic conditions therefore enhance a range of functions associated with the tumour-promoting phenotype of TAMs, at least in vitro.

### Buffering tumour-secreted acids alters TAM phenotype in vivo and reduces tumour progression

**Fig. 4:**
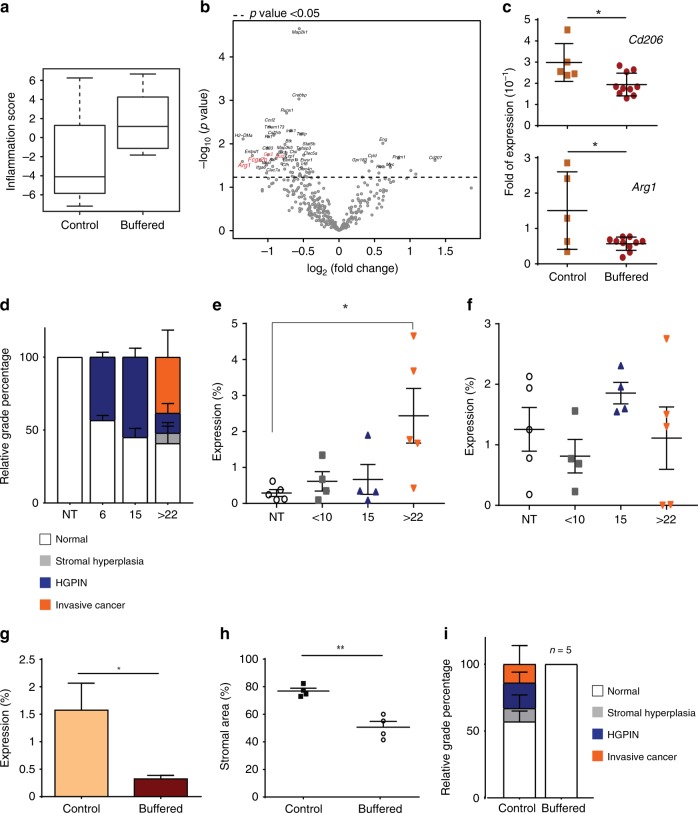
Buffering tumour-secreted acids alters tumour associated macrophage (TAM) phenotype in vivo and reduces tumour progression. **a** Inflammation score and **b** Volcano plot generated by the nSolver software 3.0 using gene expression data from nCounter PanCancer Immune profiling of CD11b^+^F4/80^+^ TAMs sorted from control or sodium bicarbonate-treated (buffered) TRAMP-C2-bearing mice (*n* = 4–5). **c** Fold expression of *Cd206* and *Arg1* in CD11b^+^F4/80^+^ TAMs sorted from independent cohort of control or sodium bicarbonate-treated (buffered) TRAMP-C2-bearing mice (*n* = 5–10). **d** Histopathological analysis of haematoxylin & eosin (H&E) samples from 6-, 15-, 22-, 23- and 25-week-old TRAMP mice. **e**, **f** Quantification of F4/80 (macrophage) and α-smooth muscle actin (fibroblast) staining in serial sections of paraffin-embedded prostates, isolated from 6-, 15-, 22-, 23- and 25-week-old TRAMP mice. **g** TRAMP mice were treated with sodium bicarbonate buffer starting from 4 weeks of age (buffered) or kept on tap water as control. F4/80-stained sections were digitally quantified and the percentage of F4/80 staining intensity in stroma of prostate tissue were plotted (*n* > 4). **h** Mean area of segmented stromal compartment (*n* = 4). **i** Histopathological analysis of H&E slides of buffered and control TRAMP mice. Data are presented as mean ± SEM. Student’s *t* test was utilised for statistical analysis; *p < 0.05, **p < 0.01

To determine whether tumour acidity was a contributing factor to the phenotype of TAMs in vivo, we treated TRAMP-C2 subcutaneously injected mice with 200 mM ad lib NaHCO_3_ as an accepted experimental approach to neutralise tumour acidity. As shown in Supplemental Fig. S4A–C, systemic sodium bicarbonate raised the intratumoural pH but with no effect on the growth of the established tumours. This provided us the opportunity to evaluate whether tumour acidity had a direct impact macrophage phenotype under constant tumour volume. In addition, analysis of myeloid cell infiltration by flow cytometry revealed no significant differences (Supplemental Fig. S4D). This provided another opportunity to test the polarising effect of acidity independent from changes in the number of immune cells. Accordingly, we then analysed the impact of buffering tumour acidity on macrophage activation using NanoString profiling and RT-PCR quantification of the selected genes in sorted TAMs. As shown in Fig. 4a, buffering tumour acidity increased the NanoString-derived “inflammation score”, denoting a shift towards a pro-inflammatory phenotype. There were also decreases in the expression of major TAMs markers, including *Arg1* and *Fcgr2b* (Fig. 4b, Supplemental Table S3). In a separate set of experiments, we also observed a significant reduction in *Cd206* and *Arg1* by single reaction RT-PCR (Fig. 4c). In agreement with this, quantitative image analysis of formalin-fixed sections showed a significant drop in the density of CD206 positivity in bicarbonate-treated tumours compared to untreated controls (Supplemental Fig. S4E). We second examined the TRAMP transgenic prostate model, which allowed us to test the effect of buffering tumour acidity over extended timescale (32 weeks). In this model, macrophage infiltration but not SMA^+^ fibroblasts corresponded with tumour progression, with the highest infiltration coincident with loss of fibromuscular tunica, disease progression from prostatic intraepithelial neoplasia lesions to high-grade adenocarcinomas and invasion (Fig. 4d–f). Algorithm-generated segmentation used to quantify those cell types is shown in Supplemental Fig. S4F. In addition, representative images are shown in Supplemental Fig. S4G. To investigate the role of pH, we treated TRAMP mice with 200 mM ad lib NaHCO_3_ for 28 weeks, starting at 4 weeks of age. Prostate tissue isolated from buffered TRAMP mice showed lower infiltration of F4/80^+^ macrophages into the stromal compartment compared to controls (Fig. 4g and Supplemental Fig. S4H). Furthermore, increasing tumour pH normalised prostate interglandular structure, decreased the relative percentage of the stromal compartment and reduced tumour incidence as compared with control (Fig. 4h, i). Together these results indicate that the acidic microenvironment contributes to the pro-tumour polarisation state of TAMs as well as tumour progression.

### Acid-responsive macrophages promote tumour growth in silico

**Fig. 5:**
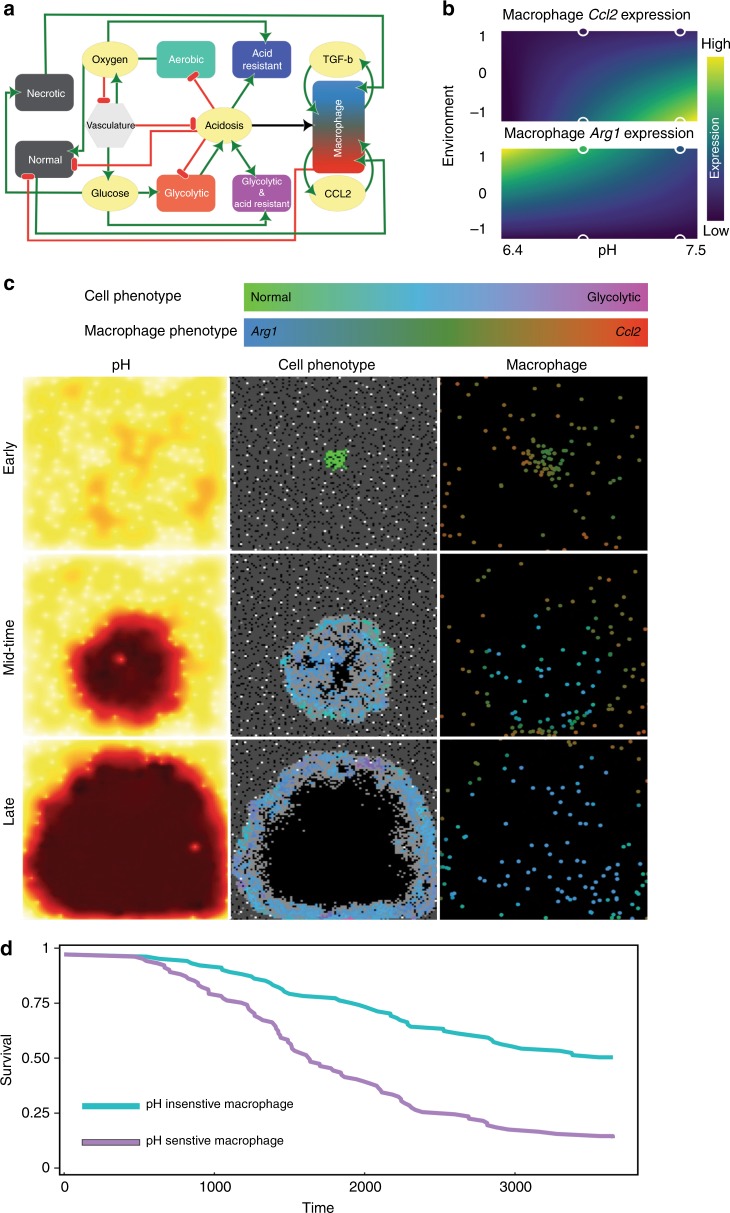
Acid-responsive macrophages promote tumour growth in silico. **a** Interaction network for agent-based model illustrating how macrophages and cells interact with, and are affected by, the microenvironment, which is composed of glucose, oxygen, acid, necrotic cells and pro- and anti-inflammatory cytokines. Green lines reflect promotion, while red lines indicate inhibitory interactions. **b** Output of linear model fitting of *Arg1* and *Ccl2* expression represented as heatmap. For each phenotypic trait, a linear model allows to predict expression under a variety of conditions. Here, −1 is a tumour-rich inflammatory environment, while 1 is environment with necrosis and anti-inflammatory cytokines. The circles outlined in white are the actual in vitro data. **c** Snapshots from agent-based model. In the pH window, low pH is dark red, while high pH is yellow. In the cell window, grey pixels are normal cells, white are vessels, and tumour cells are coloured by their phenotype. In the macrophage window, each macrophage is coloured by the mix of CCL2 and ARG1 expressed. Top panel is early in the simulation, bottom panel is when the tumour has taken >90% of the domain and the simulation is stopped. **d** Simulated survival curves generated after running the simulation under two scenarios, one hundred times each. The “pH Insensitive Macrophage” scenario is where macrophages are not affected by pH, while macrophage behaviour is affected by acid in the “pH Sensitive Macrophage” scenario. Here survival time is the amount of time it took the tumour to take >90% of the domain, given a maximum amount of time of 10 years. Mantel–Haenszel test reveals that these survival curves are significantly different, with *p* < 0.05

Despite the effect of neutralising tumour acidity on prostate carcinogenesis and its impact on the phenotype of TAMs, it was unclear whether these were functionally related, as acidic pH is thought to impact a range of other biological processes within tumours. To test whether acid-responsive macrophages can enhance tumour progression, we developed an in silico agent-based model (Fig. 5a) that allowed us to turn off macrophage acid-induced responses regardless of the underlying mechanisms and compare the responses in a heterogeneous microenvironment.

To our knowledge, this experiment can only be conducted in silico, as it is not possible to experimentally “turn off” a macrophage’s response to extracellular pH. The model complements the in vitro experiments, which were conducted in extreme and constant conditions. However, in this model, the changes in pH, cytokine concentrations and spatial co-localisation of macrophages with tumour cells creates a dynamic environment in which macrophages change phenotypes over time. This ever-changing environment thus determines macrophages’ ability, or lack thereof, to control or eradicate the tumour. Given that the model was parameterised using the experimental data, these simulations simulate the interactions in a way that is not feasible with in vitro techniques at present.

In this model, tumour acidity emerges from increased glycolytic metabolism in combination with poor perfusion, and it affects macrophage phenotype as modulated between two extremes states (Fig. 5b). Two scenarios were imposed in order to determine the impact of pH on the ability of a constant number of macrophages to modulate tumour growth. In the first scenario, macrophages behave phenotypically as if they are in pH 7.4 regardless of the actual local pH value (i.e. the value of *p* in Eq. 1 is set to 7.4 regardless of the actual local pH). In the second scenario, macrophage behaviour is modulated by setting *p* in Eq. 1 to the local pH calculated at that position in the model. Simulations were run until either the tumour took >90% of the domain or ten simulated years had elapsed, indicating that the tumour had successfully been eradicated or controlled. Each scenario was run 100 times, and the time of 90% takeover was recorded at the end of each run. As shown in representative simulation images (Fig. 5c), the extracellular acidosis, created by excess tumour glycolysis, dynamically changes the macrophage phenotype represented by the *Arg1* and *Ccl2* expression. The time to tumour takeover can be visualised using Kaplan–Meier curves (Fig. 5d). The tumours grew much more rapidly in the simulations where acidosis was actively modulating macrophage behaviour. The difference in these survival curves was significant, with *p* < 0.001, as calculated using Mantel–Haenszel test. The results from these simulations suggest that acid released by tumour cells can create a protective niche capable of directing the functional role of macrophages, thereby increasing tumour growth and decreasing time to progression.

## Discussion

Tumours undergo metabolic transformation that rewires cellular metabolism to promote tumourigenicity, immune evasion and disease recurrence.^39^ One of these metabolic abnormalities is upregulation of glycolysis, even under aerobic conditions. High rate of glycolysis provides malignant cells with proliferative privilege by facilitating uptake and incorporation of nutrients into the growing biomass.^40^ Metabolic by-products of glycolysis, such as lactic acid, also cause a heterogeneous acidification of the extracellular space, which can results in immunosuppressive nature of the TME.^6,41^ Unlike studies that combine lactate and H+ ions as single functional entity named “lactic acid”, we identified an independent role of tumour-generated acidity in driving TAMs phenotype, which in turn can contribute to tumour progression.

In the current investigation, we propose a scenario in which acids generated by glycolytic cells alter the phenotype of TAMs, creating a permissive niche for cancer progression in prostate cancer. Using zwitterionic organic chemical buffering system, our data show that acidic pH alter the activation state of macrophages incubated under polarising conditions, directing the cells towards a functional state similar to the pro-tumour phenotype often ascribed to TAMs. Furthermore, we demonstrate that buffering tumour acidosis alters the activation state of TAMs, with a significant reduction in genes such as *Arg1* and *Cd206* that are usually associated with a tumour-promoting role for this population. Finally, we noted an association between tumour progression, acidosis and the presence of macrophages in prostate cancer progression in mice and human disease and utilise an in silico agent-based model to delineate a role for acidosis in regulating macrophage phenotype and tumour progression. Cumulatively, these results suggest that tumour acidosis is an important factor that dictates the pro-tumour functionality of macrophages in prostate cancer.

Lactic acid produced by tumour cells was reported earlier to polarise macrophages into an M2-like phenotype, with *Arg1* expression by macrophages essential for lung cancer and melanoma growth.^15^ In addition, Carmona-Fontaine et al. have demonstrated that lactate cooperates with hypoxia to induce the expression of ARG1 in macrophages. Through the employment of an agent-based model, they also showed that hypoxia-responsive macrophages induce faster tumour growth.^42^ However, there is limited information regarding how acidity, independent from those metabolic factors, can influence properties of macrophages in TME. Only recently, Toszka et al. identified a role of tumour acidity independent from lactate in driving growth of melanoma cell line B16 in cAMP-dependent manner.^43^ In the current study that investigates prostate cancer, we also provide evidence that acidic pH, independent from lactate, can promote the pro-tumour polarisation of macrophages, including enhanced tumour cell proliferation, loss of cytotoxicity and release of angiogenic factors. In silico modelling also demonstrated that modulation of the macrophage phenotype by acidity was a significant driver of tumour progression and immune suppression.

### Future directions and translational impact

Acidic TME is a promising target for tumour-specific imaging and therapy. For example, pH-responsive peptides and pH-sensitive nanotechnology-based systems were shown to improve the efficacy and specificity of cancer therapeutics and diagnostics.^44–46^ An immuno-conjugate that integrates urease enzyme with the ability of pH alkalisation in TME is currently undergoing clinical trials in lung cancer but has not been tested in prostate cancer.^47,48^ Despite the efficacy of those modalities evident in preclinical studies, no studies had been conducted to investigate their impact on TME. Among the many future therapeutic applications of the current study is to test whether pH-sensitive macrophage-specific immuno-conjugates or nano-systems that specifically target acidic areas rich in macrophages can reduce immunosuppression and increase T cell infiltration. Those approaches hold the promise of improving efficacy of T cell immunotherapeutic strategies in prostate cancer.

## Supplementary information

Supplemental Information

## Data Availability

The authors declare that the main data supporting the findings of this study are available within the article and its Supplementary Information files. Mathematical model code is available for access on github https://github.com/MathOnco/macrophage_pH.
