# Correction: Acidity promotes tumour progression by altering macrophage phenotype in prostate cancer

**DOI:** 10.1038/s41416-019-0710-4

**Published:** 2020-01-14

**Authors:** Asmaa El-Kenawi, Chandler Gatenbee, Mark Robertson-Tessi, Rafael Bravo, Jasreman Dhillon, Yoganand Balagurunathan, Anders Berglund, Naveen Vishvakarma, Arig Ibrahim-Hashim, Jung Choi, Kimberly Luddy, Robert Gatenby, Shari Pilon-Thomas, Alexander Anderson, Brian Ruffell, Robert Gillies

**Affiliations:** 1 Department of Pharmacology and Toxicology, Faculty of Pharmacy, Mansoura University, Mansoura, Egypt; 2 Department of Immunology, H. Lee Moffitt Cancer Center, Tampa, FL 33612 USA; 3 Department of Cancer Physiology, H. Lee Moffitt Cancer Center, Tampa, FL 33612 USA; 4 Department of Integrated Mathematical Oncology, H. Lee Moffitt Cancer Center, Tampa, FL 33612 USA; 5 Department of Anatomic Pathology, H. Lee Moffitt Cancer Center, Tampa, FL 33612 USA; 6 Department of Biostatistics, H. Lee Moffitt Cancer Center, Tampa, FL 33612 USA; 7 Department of Radiology, H. Lee Moffitt Cancer Center, Tampa, FL 33612 USA; 8 Department of Breast Oncology, H. Lee Moffitt Cancer Center, Tampa, FL 33612 USA

**Keywords:** Immune evasion, Cancer microenvironment

**Correction to:** *British Journal of Cancer* **121**, 556–566; 10.1038/s41416-019-0542-2, published online 16 August 2019

The author Naveen Vishvakarma was incorrectly listed as Naveen Visvakarma. The correct name is listed above.

